# Assessment of various continual reassessment method models for dose-escalation phase 1 oncology clinical trials: using real clinical data and simulation studies

**DOI:** 10.1186/s12885-020-07703-6

**Published:** 2021-01-05

**Authors:** G. D. James, S. Symeonides, J. Marshall, J. Young, G. Clack

**Affiliations:** 1 Medical Statistics Consultancy Ltd, London, W4 5XF UK; 2 Edinburgh Cancer Research Centre, IGMM, University of Edinburgh, Edinburgh, EH4 2XU UK; 3 Oncology Biometrics, Oncology R&D, AstraZeneca, Cambridge, UK; 4 Aptus Clinical Ltd, Alderley Park, Macclesfield, Cheshire SK10 4TF UK; 5 Department of Oncology and Metabolism, University of Sheffield, Sheffield, UK

**Keywords:** Clinical trial, Phase 1, Continual reassessment method, Skeleton, Bayesian, Oncology

## Abstract

**Background:**

The continual reassessment method (CRM) identifies the maximum tolerated dose (MTD) more efficiently and identifies the true MTD more frequently compared to standard methods such as the 3 + 3 method. An initial estimate of the dose-toxicity relationship (prior skeleton) is required, and there is limited guidance on how to select this. Previously, we compared the CRM with six different skeletons to the 3 + 3 method by conducting post-hoc analysis on a phase 1 oncology study (AZD3514), each CRM model reduced the number of patients allocated to suboptimal and toxic doses. This manuscript extends this work by assessing the ability of the 3 + 3 method and the CRM with different skeletons in determining the true MTD of various “true” dose-toxicity relationships.

**Methods:**

One thousand studies were simulated for each “true” dose toxicity relationship considered, four were based on clinical trial data (AZD3514, AZD1208, AZD1480, AZD4877), and four were theoretical. The 3 + 3 method and 2-stage extended CRM with six skeletons were applied to identify the MTD, where the true MTD was considered as the largest dose where the probability of experiencing a dose limiting toxicity (DLT) is ≤33%.

**Results:**

For every true dose-toxicity relationship, the CRM selected the MTD that matched the true MTD in a higher proportion of studies compared to the 3 + 3 method. The CRM overestimated the MTD in a higher proportion of simulations compared to the 3 + 3 method.

The proportion of studies where the correct MTD was selected varied considerably between skeletons. For some true dose-toxicity relationships, some skeletons identified the true MTD in a higher proportion of scenarios compared to the skeleton that matched the true dose-toxicity relationship.

**Conclusion:**

Through simulation, the CRM generally outperformed the 3 + 3 method for the clinical and theoretical true dose-toxicity relationships. It was observed that accurate estimates of the true skeleton do not always outperform a generic skeleton, therefore the application of wide confidence intervals may enable a generic skeleton to be used. Further work is needed to determine the optimum skeleton.

## Background

The continual reassessment method (CRM) is more efficient for estimating the recommended dose from phase 1 clinical trials for further development than the commonly used 3 + 3 method. In addition the 3 + 3 method leads to more patients than necessary receiving suboptimal doses ([1, 2] and it has limitations in its ability to correctly identify the maximum tolerated dose (MTD). Studies have found that, compared to the 3 + 3 method, the CRM allocates fewer patients to suboptimal [3] and harmful doses ([2, 4] and identifies the true MTD a higher proportion of the time ([5, 6], reducing the likelihood of making a costly and potentially unsafe decision.

The use of model-based methods including the CRM in phase 1 trials is low. Literature reviews have found model-based methods were used in 3.3% of phase 1 trials between 2007 and 2008 [7], and 1.6% of trials between 1991 and 2006 [8]. A lack of practical guidance for implementing these methods [9], and increased statistical complexity (e.g. applying a complicated” black box” algorithm [10]) may be preventing uptake of these methods. The CRM requires pre-specification of the dose-toxicity model, which consists of initial estimates of the probability of experiencing a DLT for each dose (prior skeleton) and a prior distribution to describe the underlying confidence in these prior probabilities [9]. The prior distribution is described elsewhere as it has been investigated previously [11]. Skeleton probabilities can be estimated using prior knowledge of the dose-toxicity relationships from pre-clinical or clinical studies [12]. However, when prior knowledge is unavailable or there is concern about how reliable the preclinical data is for estimating the skeleton, the choice of the probabilities in the skeleton is a challenge ([12, 13] and may not be accurate [7]. We found limited guidance on a standard skeleton to be used when there is limited knowledge on dose-toxicity relationship, which is an area of need to support practical implementation of CRM. An approach that deserves some consideration is using indifference intervals to determine prior probabilities [9]. Also, O’Quigley and Iasonos [14] investigated the relationship between priors and skeletons.

A common extension of the CRM, is the extended (or two-stage) CRM [2], which has the advantage of ensuring the first patients are recommended to receive the lowest dose. The first stage involves enrolling up to three patients in each cohort, starting with the lowest dose and escalating one dose each time until the first DLT is experienced. In the second stage, the CRM method is updated based on toxicity data and is used to recommend the next, and all further dose levels to be tested.

We proposed 6 different skeletons for use in the CRM when there is limited information about the dose-toxicity relationship, and post-hoc analysis on AZD3514 showed the CRM with each skeleton identified the true MTD and reduced the number of patients allocated to suboptimal and toxic doses compared to the 3 + 3 method [15]. However, the ability of these methods has only been assessed for one clinical dose-toxicity curve, therefore it would be useful to assess their performance in various dose-toxicity relationships to contribute to guidance on skeletons for the CRM model. The previous manuscript compared skeletons based on the number of patients who received optimal and suboptimal doses using a single sample of the true dose-toxicity curve. This research assesses the ability of each skeleton to determine the correct MTD for multiple true dose-toxicity curves. Furthermore, we use simulation so we sample multiple data from each true dose-toxicity curve generating a range of trial data which could not be achieved from a single study which provides a more generally applicable assessment of performance of the different approaches in determining the true MTD.

We sought to compare the ability of the Extended-CRM with Bayesian design, using different skeletons, with the 3 + 3 method to identify the MTD. “True” clinical dose-toxicity curves were identified from four phase 1 clinical trials: AZD3514, AZD1208, AZD1480 and AZD4877, and four additional theoretical true dose-toxicity curves were constructed. Simulations studies were conducted to identify the proportion of simulations where the true MTD was identified correctly, underestimated and overestimated using each method. In this manuscript we present the results of this analysis and provide recommendations in the discussion to improve the uptake of these methods. This research has been presented externally at PSI [16] and PSI [17].

## Methods

The highest 5 doses from four phase 1 clinical trials were used. AZD3514 study 1 (NCT01162395) has been described before [15]. AZD1208 study 1 (NCT01489722) consisted of patients with recurrent or refractory Acute Myelogenous Leukemia (AML) being given AZD1208, a novel agent that inhibits Proviral Integration Moloney virus (PIM) kinases 1, 2 and 3. Patients received doses of AZD1208 monotherapy of 120 mg QD, 240 mg QD, 480 mg QD, 700 mg QD, and 900 mg QD. The highest dose was considered intolerable, but no MTD was determined as no dose cohort met the criteria of 6 patients evaluable for DLT. AZD1480 study 2 (NCT01219543) consisted of patients with advanced solid malignancies and estimated glomerular filtration rate or ROS-mutant Non-small cell lung cancer or non-smokers with lung metastasis. Patients received doses of 20 mg BID, 30 mg BID, 35 mg BID and 45 mg BID. To extend the range of dose levels for the dose toxicity relationship for AZD1480, an additional dose level, 15 mg AZD1480 BID from AZD1480 study 4 (NCT01219543), was also included. AZD4877 study 6 (NCT00471367) consisted of patients with advanced solid malignancies including lymphoma. Patients received twice weekly doses of 2 mg, 4 mg, 7 mg, 11 mg and 15 mg.

The number and proportion of patients who experienced a DLT, by dose within each study is presented in Table 1. For each dose we considered the observed proportion of patients who experienced a DLT as the true dose-toxicity, unless this proportion is lower than the dose below, in which case the proportion will be the same as this dose. This formed 4 unique clinical dose-toxicity curves, representative of what was observed in the clinic for these studies. Notably, the second and third dose of AZD1480 has the same probability of toxicity, which is unusual, as we expect the probability of DLT to increase with increasing dose.

**Table 1:** Dose-toxicity relationship for each clinical study

Study	Dose	DLTs	Number of patients evaluable for DLT	Proportion of patients who experienced a DLT	P (DLT) for true dose toxicity curve
AZD3514	250 mg QD	0	6	0%	0%
	500 mg QD	0	6	0%	0%
	1000 mg QD	1	6	17%	17%
	1000 mg BID	3	6	50%	50%
	2000 mg BID	4	4	100%	100%
AZD1208	120 mg QD	0	3	0%	0%
	240 mg QD	0	3	0%	0%
	480 mg QD	0	3	0%	0%
	700 mg QD	1	4	25%	25%
	900 mg QD	2	3	67%	67%
AZD1480	15 mg BID^a^	0	4	0%	0%
	20 mg BID	1	5	20%	20%
	30 mg BID	1	5	20%	20%
	35 mg BID	2	3	67%	67%
	45 mg BID	0	2	0%	67%
AZD4877	2 mg twice weekly	0	3	0%	0%
	4 mg twice weekly	0	3	0%	0%
	7 mg twice weekly	0	3	0%	0%
	11 mg twice weekly	0	6	0%	0%
	15 mg twice weekly	2	2	100%	100%

^a^Taken from another study on AZD1480

BID: twice daily; QD: once daily

**Fig. 1:**
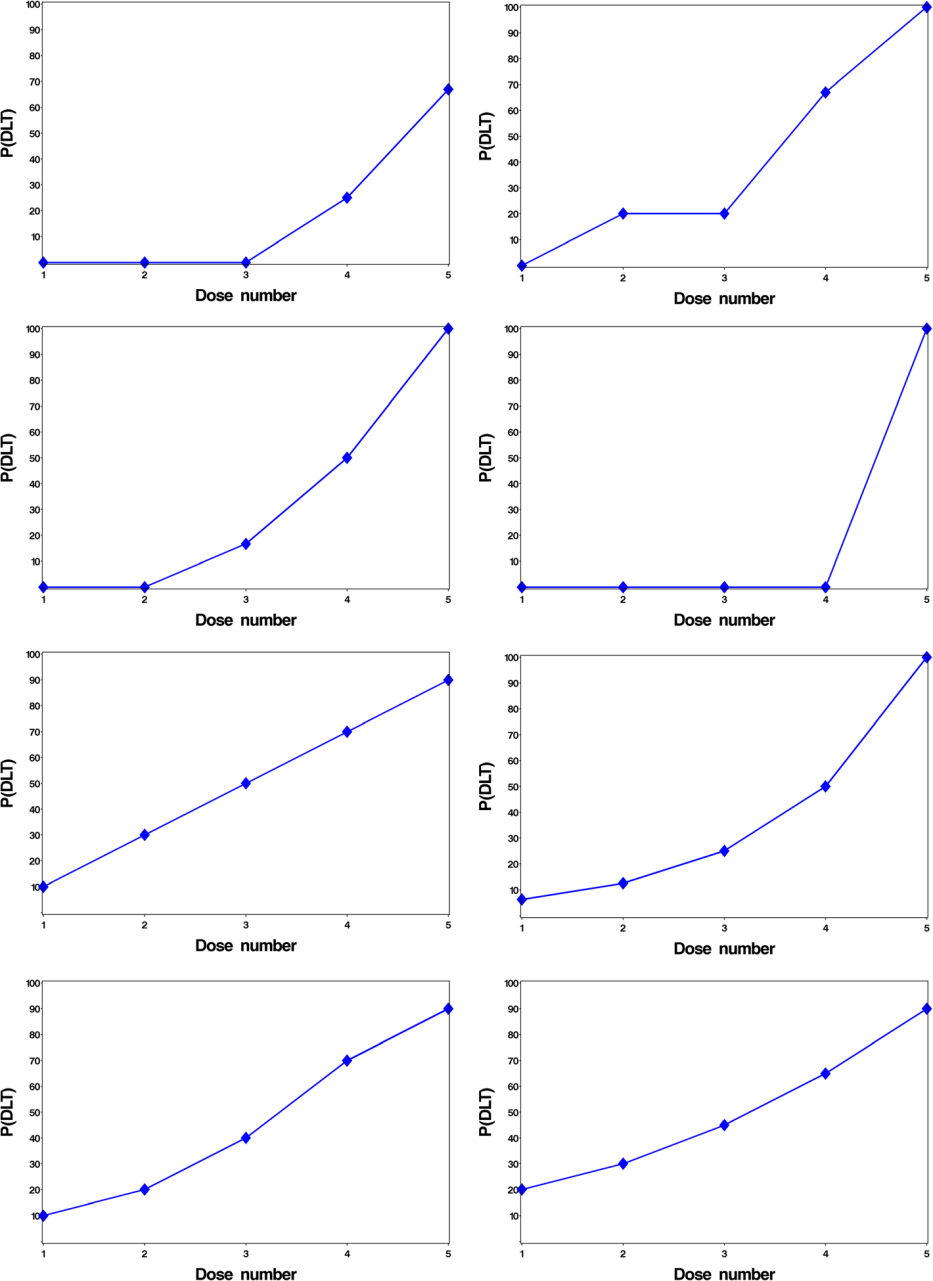
True dose toxicity curves individually

In addition, four theoretical true dose-toxicity curves were used: conservative, step-up, dose-linear and sigmoidal, which are taken from skeletons used in our previous study [15]. For this simulation study, the choice of 4 theoretical dose toxicity curves from the 8 presented in [15] were selected to ensure a range of dose toxicity relationships were included in the analysis. The eight true dose toxicity curves are presented in Fig. 1.

**Fig. 2:**
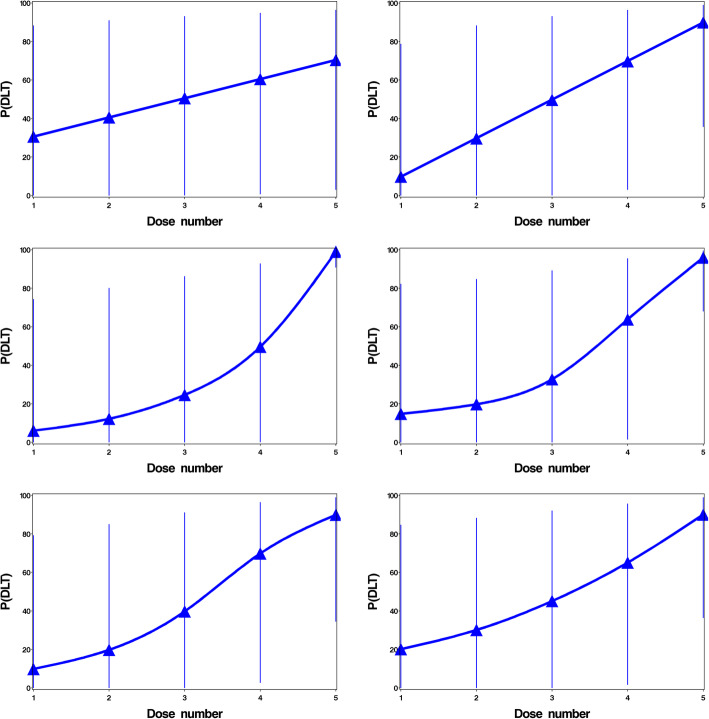
CRM skeletons and 95% prediction intervals

The CRM uses a Bayesian model which assumes increasing probability of DLT with increasing dose [12]. It consists of 3 components: the dose toxicity model, skeleton and prior distribution, which are explained in detail in [15]. Also, the maximum acceptable toxicity level (maximum acceptable proportion of patients experiencing a DLT), must be specified during the design of the study. The skeleton is formed by specifying initial estimates of probabilities of DLT at each dose. It is continually updated as new toxicity information emerges: a DLT experienced at the current dose level would increase the estimated probability of experiencing a DLT at the current dose, and all higher doses; no DLT experienced at the current dose level would reduce the estimated probability of DLT at all dose levels. After the model is updated, it will recommend that the next patient(s) are allocated the dose which is closest to but below the maximum acceptable toxicity level. Dose-skipping is an increase from the current dose by more than 1 dose level. We chose to not allow dose-skipping, as in clinical practice it is unlikely that this would be allowed due to an increased safety risk. We consider six skeletons; conservative, aggressive, step-up, dose-linear, sigmoidal, and O’Quigley. The rationale for these skeletons are described and analysed in [15], and are displayed in Fig. 2.

The maximum acceptable toxicity level of the 3 + 3 level is generally assumed to be 33% [18], but is debated in the literature as being between 17 and 33%. Wheeler et al [19] introduced the concept of a tipping point in A + B designs in which they conclude that the selected MTD in a 3 + 3 design is most likely to have a true probability ≤29.7%.

The focus of this manuscript is to evaluate the correct determination of the MTD for each method, however it is noted that sample size is also an important practical consideration when conducting a clinical trial. Simulation studies have shown that the CRM and 3 + 3 method are comparable in terms of sample size needed to determine the MTD when testing ≤5 doses [5], therefore it is reasonable to expect similar sample sizes for the 3 + 3 and CRM method in these simulations.

### Statistical analysis

The maximum acceptable toxicity level was set at 33% to aid comparison with the 3 + 3 method. Therefore, for each true dose-toxicity curve, the MTD was the highest dose where the probability of DLT was below or equal to 33%. For each true dose-toxicity curve, we simulated 1000 dose-toxicity datasets, and used the 3 + 3 method and the extended CRM method with six different skeletons to identify the MTD using EAST [20]. For each method we calculated the percentage of simulated studies where the correct MTD was identified. For the extended CRM method, if a simulation at any time during the second stage of the method (CRM allocates next dose) has a mean predicted posterior probability of experiencing a DLT at the lowest dose that exceeds the maximum acceptable toxicity, the simulation would stop and not be able to find an MTD. For the 3 + 3 model, if 2 or more DLTs were experienced at the lowest dose of a simulation the MTD could not be determined.

The extended CRM models used a single parameter logistic model to model the dose-toxicity relationship, with a Gaussian prior distribution for the natural logarithm of the logistic model parameter of mean 0 and variance 1.34 for each skeleton. The variance was chosen to be 1.34 as with other published analyses [9].

For each model, we considered six skeletons; conservative, aggressive, step-up, dose-linear, sigmoidal, and O’Quigley which are displayed in Fig. 2a. Prior to the CRM being used to determine the next recommended dose, the Goodman’s modification was used with cohorts of 3 patients to match the 3 + 3 method for comparison. After this, the CRM model was updated after each individual patient received treatment (cohorts of 1 patient). Dose-skipping was not allowed.

The maximum number of patients to be exposed to a single dose level was set to be six which gives a maximum sample size of 30, given a maximum number of 5 cohorts. Therefore, when the model recommends a 7th patient be assigned to a single dose level, the study will end, and the MTD will be determined as the dose with the largest estimated probability of DLT below or equal to the maximum acceptable toxicity level (33%). No other stopping criteria were included in the simulations. This aids comparison with the 3 + 3 method, because another dose may be explored after six patients, but no more than 6 patients would be in a single cohort.

## Results

Table 2 (section A) displays the proportion of simulations where the MTD was correctly identified for each dose-escalation method on the ‘clinical trial dose toxicity curves’. All methods correctly identified the MTD for AZD4877. Each CRM model correctly identified the MTD more frequently than the 3 + 3 method for studies AZD1208 and AZD1480. The ability of the CRM to correctly identify the MTD for AZD3514 varied considerably, varying from 34.8 to 71.7%, of which the aggressive (34.8%) and dose-linear (36.4%) skeletons were particularly poor. Notably for AZD3514, only two of the six CRM skeletons (conservative and sigmoidal) identified the correct MTD a higher proportion of the time than the 3 + 3 method. The percentage of simulations where the correct MTD was identified using the conservative and sigmoidal skeletons was fairly consistent across studies AZD1208, AZD1480 and AZD3514, but the other CRM skeletons varied more across studies. Specifically, the percentage of simulations where the correct MTD was identified varied between 36.4 and 98.5% for the dose-linear skeleton, and 34.8 and 88.3% for the aggressive skeleton.

**Table 2:** Proportion of simulations where true MTD identified

**A. Clinical trial dose toxicity curves**					
**Dose escalation method**		**True dose toxicity curve**			
		**AZD3514**	**AZD1208**	**AZD1480**	**AZD4877**
**3 + 3**		64.9	*50.5*	*45.5*	100.0
**Extended CRM**	**Conservative**	***71.7***	66.8	60.9	100.0
	**Aggressive**	*34.8*	88.3	51.0	100.0
	**Step-up**	57.6	88.9	60.1	100.0
	**Dose-Linear**	36.4	***98.5***	60.4	100.0
	**Sigmoidal**	71.4	66.8	***68.4***	100.0
	**O’Quigley**	56.5	78.2	59.8	100.0
**B. Theoretical dose toxicity curves**					
**Dose escalation method**		**True dose toxicity curve**			
		**Conservative**	**Step-up**	**Dose-linear**	**Sigmoidal**
**3 + 3**		*33.1*	*22.7*	*36.8*	46.6
**Extended CRM**	**Conservative**	***63.4***	**43.9**	53.9	**50.3**
	**Aggressive**	44.6	30.8	48.2	33.9
	**Step-up**	44.5	29.8	64.2	32.6
	**Dose-Linear**	39.7	23.6	62.7	*22.8*
	**Sigmoidal**	51.7	33.0	**67.8**	33.2
	**O’Quigley**	40.4	27.3	65.9	28.9

The method which correctly identified the MTD of a true dose toxicity curve in the greatest percentage of simulations is italicised and emboldened. The method which correctly identified the MTD of a true dose toxicity curve in the lowest percentage of simulations is italicised and underlined

Table 2 (section B) displays the proportion of simulations where the MTD was correctly identified for each method on the ‘theoretical dose toxicity curves’. Each CRM skeleton identified the true MTD more frequently for the conservative, step-up and dose-linear true dose toxicity curves compared to the 3 + 3 model. However, only the conservative CRM skeleton correctly identified the MTD more frequently than the 3 + 3 method for the sigmoidal dose-toxicity curve. Comparing CRM models only, the conservative skeleton identified the correct MTD in a higher proportion of simulations for the conservative, step-up and sigmoidal dose-toxicity curves, but not the dose-linear dose-toxicity curve. Using the CRM with initial skeleton DLT probabilities matching the true dose toxicity curve did not always produce the best results; for example the conservative skeleton more frequently identified the true MTD of the sigmoidal true dose toxicity curve than the sigmoidal skeleton.

Table 3 shows the proportion of simulations where the MTD was correctly identified, underestimated, overestimated or not determined, averaged over the clinical trial dose-toxicity curves (A), theoretical dose-toxicity curves (B), and all dose-toxicity curves (C). It is worth noting that, on average, each method correctly identified the MTD more frequently in the clinical trial dose toxicity curves compared with the theoretical dose toxicity curves. Each CRM skeleton identified the true MTD more frequently for the clinical trial, theoretical and all dose toxicity curves compared to the 3 + 3 method. The percentage point increase in correct identification of the MTD for the CRM skeleton compared to the 3 + 3 method ranged from 3.3 to 12.5 for clinical trial dose-toxicity curves, 2.4 to 18.1 for theoretical dose-toxicity curves and 3.9 to 13.9 for all dose-toxicity curves. Compared to the 3 + 3 method, each CRM skeleton was more than twice as likely to overestimate the MTD and less than half as likely to underestimate the MTD. The MTD could be determined in over 99% of simulations for clinical trial dose-toxicity curves using each method, but could not be determined for the theoretical dose-toxicity curves for approximately 5% of simulations using the CRM skeleton and 15% of 3 + 3 simulations.

**Table 3:** Summary statistics about each dose escalation method

**A. Clinical trials** dose toxicity curves					
**Dose escalation method**		**MTD selected by dose-escalation method (%)**			
		**True selected**	**Underestimated**	**Overestimated**	**< lowest dose**
**3 + 3**		65.2	31.8	2.3	0.7
**Extended CRM**	**Step-up**	76.7	9.0	13.9	0.4
	**Dose-Linear**	76.7	4.2	21.5	0.4
	**O’Quigley**	76.6	11.6	14.4	0.4
	**Conservative**	74.8	16.6	8.2	0.4
	**Sigmoidal**	73.8	14.7	8.3	0.4
	**Aggressive**	68.5	6.7	24.4	0.4
**B. Theoretical dose toxicity curves**					
**Dose escalation method**		**MTD selected by dose-escalation method (%)**			
		**True selected**	**Underestimated**	**Overestimated**	**< lowest dose**
**3 + 3**		34.8	42.6	7.3	15.3
**Extended CRM**	**Conservative**	52.9	21.6	20.7	4.9
	**Sigmoidal**	46.4	19.1	29.9	4.6
	**Step-up**	42.8	22.1	30.1	5.1
	**O’Quigley**	40.6	21.4	32.9	5.1
	**Aggressive**	39.4	21.3	34.5	4.9
	**Dose-Linear**	37.2	18.3	40.4	4.1
**C. All dose toxicity curves**					
**Dose escalation method**		**MTD selected by dose-escalation method (%)**			
		**True selected**	**Underestimated**	**Overestimated**	**< lowest dose**
**3 + 3**		50.0	37.2	4.8	8.0
**Extended CRM**	**Conservative**	63.9	19.1	14.4	2.6
	**Sigmoidal**	61.5	16.9	19.1	2.5
	**Step-up**	59.7	15.5	22.0	2.8
	**O’Quigley**	57.1	16.5	23.6	2.7
	**Dose-Linear**	55.5	11.3	31.0	2.2
	**Aggressive**	53.9	14.0	29.4	2.6

The aggressive skeleton was the skeleton with the lowest mean proportion of simulations identifying the true MTD for the clinical trial dose-toxicity curves (68.5%), the other CRM methods had similar proportions to each other (73.8 to 76.7%). For the theoretical dose-toxicity curves, there was more variation between the CRM skeletons in the mean proportion of simulations where the true MTD was identified. The highest percentage was for the conservative skeleton (52.9%), followed by the sigmoidal skeleton (46.4%). Similarly, for all dose-toxicity curves, there was some variation in the average percentage of simulations where the true MTD was selected, and the highest frequency was by the conservative and sigmoidal skeletons. The conservative skeleton was the skeleton in the CRM method which overestimated the MTD least frequently. The dose-linear and aggressive skeletons overestimated the MTD considerably more frequently than other skeletons, however they also underestimated the MTD somewhat less frequently than other skeletons.

## Discussion

This analysis compared the extended CRM, using various skeletons, with the 3 + 3 method for clinical and theoretical ‘true’ dose-toxicity scenarios. The results provide further support that the CRM is more likely to identify the true MTD compared to the 3 + 3 method. It is notable that the CRM less frequently underestimated and more frequently overestimated the MTD compared to the 3 + 3 method, providing evidence the 3 + 3 method is more conservative. This overestimation can be mitigated by adapting the CRM method [21]. The choice of skeleton has significant impact on the likelihood of overestimating or underestimating the MTD, and this should be taken into consideration when planning dose-escalation trials using this method and highlights the importance of simulating the operating characteristics for the dose escalation during the design stage.

To our knowledge this is the first study to compare skeletons for the CRM method in a variety of dose-toxicity relationships. It builds on our previous analysis, where the CRM method with various skeletons was applied retrospectively to clinical trial data from AZD3514 study 1, which found that the CRM method required fewer patients to identify the true MTD and allocated fewer patients to sub-optimal and toxic doses [15]. The majority of studies implementing the CRM method uses the skeleton from O’Quigley et al [22] without providing justification [9]. In our literature review we found the majority of dose-escalation studies did not provide the skeletons or explain how they were obtained.

When taking into account all of the simulations, the CRM estimated the true MTD more frequently than the 3 + 3 method. The CRM overestimated the true MTD more frequently than the 3 + 3 method. One probable reason for this difference is that the 3 + 3 method actually has maximum acceptable toxicity of between 17% (1 out of 6 patients) and 33% (2 out of 6 patients). The choice of skeleton can be used to reduce the chances of overestimation of the MTD and further lowered by reducing the prior probabilities in each the skeleton, although this may cause slower dose-escalation requiring more patients to identify the MTD and exposing more patients to a potentially toxic drug or a sub-optimal dose. James et al describes how clinical opinion should also be used in conjunction with the CRM recommendation for determining the next dose [15], which improves the flexibility of dose choice and could also prevent the MTD being overestimated.

The CRM and 3 + 3 method identified the true MTD more frequently in the clinical trial dose toxicity curves compared to the theoretical dose-toxicity curves. One likely reason for this is the dose toxicity curves from the clinical trials curves are steeper and often the dose immediately greater than the MTD has a considerably greater probability of toxicity. For instance, the dose immediately above the MTD has probability of toxicity of 50% or greater for all the clinical trial dose-toxicity curves, whereas two of the four theoretical dose-toxicity curves have this probability at less than 50%.

There was little difference between the skeletons in the average percentage of MTDs correctly identified for the clinical trial dose-toxicity curves. However, there were considerable differences between skeletons in the average percentage of MTDs correctly identified for the theoretical dose-toxicity curves. Conservative and sigmoidal skeletons correctly identified the MTD in approximately half of the simulations, aggressive and dose-linear least frequently identified the MTD correctly identified in just under 40% of simulations. Looking closer at the aggressive and dose-linear skeletons, their ability to identify the true MTD varies more between dose-toxicity curves compared to the other skeletons. They were particularly poor at identifying the true MTD in AZD3514 with correct identification in just over a third of simulations, which was considerably less than the other skeletons and the 3 + 3 design. However, the dose-linear skeleton identified the correct MTD in study AZD1208 more frequently than any other skeleton. The conservative method was the least variable skeleton in terms of percentage of correct MTD identification. Notably all methods identified the true MTD of the theoretical Sigmoidal dose-toxicity-curve in 50% of fewer simulations, on closer inspection this is likely due to the dose above the true MTD having a 35% probability of DLT, which is just above the 33% maximum acceptable toxicity level.

The choice of skeleton had considerable influence on the frequency of simulations underestimating or overestimating the MTD. The conservative and sigmoidal skeletons had the lowest frequency of overestimations, and the highest frequency of underestimations, suggesting these may be the most conservative methods. The dose-linear and aggressive skeletons had the highest frequency of overestimations and lowest frequency of underestimations, suggesting these may be the least conservative methods.

Notably starting with a skeleton which matched the true dose-toxicity curve did not guarantee it was the skeleton most likely to identify the MTD. For instance, the conservative skeleton identified the true MTD of the step-up dose-toxicity curve more frequently than the step-up skeleton. This suggests that it may be less important to accurately specify a skeleton that matches the true dose toxicity relationship during the design of a study however to understand the characteristics of a chosen skeleton, simulation is advised. One alternative approach which should be considered is conducting the CRM on multiple skeletons simultaneously and taking a Bayesian model averaging (BMA) approach as proposed by Yin Y & Yuan Y [13], where a recommendation for how to apply skeletons can be described by Haitao P & Yuan Y [23].

### Strengths limitations

This study has several strengths. It uses dose-toxicity curves from four compounds from phase 1 clinical trials where patients were allocated one of 5 or more doses, and information on patient characteristics were available. It also uses four theoretical dose-toxicity curves which were derived from physician’s beliefs about possible relationships between dose and toxicity. EAST, advanced statistical software was utilised to conduct simulation studies to assess the ability of the CRM, with various skeletons and the 3 + 3 method to identify the true MTD for these dose-toxicity curves. A limitation of this study is that we could not incorporate clinical opinion as part of the simulations. Dose levels do not all have the same multiplicative increments and intermediate dose levels may not have been specified apriori but this analysis utilised real world data so was based on those retrospective dose levels.

### Implications

This research has implications for planning and conducting future phase I oncology dose escalation trials. Simulations support the existing literature that the CRM more often correctly identifies the true MTD compared to the 3 + 3 design. The importance of selecting an appropriate skeleton and understanding its characteristics for different underlying truths has been shown, and should support the study objectives. Several skeletons have been proposed, and compared using a variety of true dose toxicity relationships, and suggestions have been made for their use in future trials.

## Conclusions

Generally, the CRM method more frequently identifies the true MTD compared to the 3 + 3 method, even when the optimal dose-toxicity curve is unknown. Choice of the skeleton should depend on study objectives. This manuscript describes skeletons that may be used where there is limited data available to describe dose-toxicity relationship and raises the importance of further exploration into this. We advise investigators who are using CRM methods to make available their initial priors and final dose-toxicity graphs so optimal generic graphs can be derived and to support the uptake of these methods.

## Data Availability

The datasets supporting the conclusions of this article are unavailable for access.

## Abbreviations

AML: Acute myelogenous leukemia
BMA: Bayesian model averaging
CRM: Continual reassessment method
DLT: Dose limiting toxicity
MTD: Maximum tolerated dose
PIM: Proviral Integration Moloney virus
